# Frailty and pre-frailty in cardiac surgery: a systematic review and meta-analysis of 66,448 patients

**DOI:** 10.1186/s13019-021-01541-8

**Published:** 2021-06-25

**Authors:** Jessica Avery Lee, Bobby Yanagawa, Kevin R. An, Rakesh C. Arora, Subodh Verma, Jan O. Friedrich, on behalf of the Canadian Cardiovascular Surgery Meta-Analysis Working Group

**Affiliations:** 1 Division of Cardiac Surgery, University of Toronto, 30 Bond Street, 8th Floor, Bond Wing, Toronto, ON M5B 1W8 Canada; 2 Department of Surgery, Max Rady College of Medicine, University of Manitoba, Winnipeg, Manitoba Canada; 3 Critical Care, St Michael’s Hospital, University of Toronto, Toronto, Ontario Canada

**Keywords:** Frailty, Coronary artery bypass graft, Valve surgery

## Abstract

**Background:**

The burden of frailty on cardiac surgical outcomes is incompletely understood. Here we perform a systematic review and meta-analysis of studies comparing frail versus pre-frail versus non-frail patients following cardiac surgery.

**Methods:**

We searched MEDLINE and EMBASE databases until July 2018 for studies comparing cardiac surgery outcomes in “frail”, “pre-frail” and “non-frail” patients. Data was extracted in duplicate. Primary outcome was operative mortality.

**Results:**

There were 19 observational studies with 66,448 patients. Frail patients were more likely female (risk ratio [RR]1.7; 95%CI:1.5–1.9), older (mean difference: 2.4; 95%CI:1.3–3.5 years older) with greater comorbidities and higher STS-PROM. Frailty (RR2.35; 95%CI:1.57–3.51; *p* < 0.0001) and pre-frailty (RR2.03; 95%CI:1.52–2.70; *p* < 0.00001) were associated with increased operative mortality compared with non-frail patients. Frailty was also associated with greater risk of prolonged hospital stay (RR1.83; 95%CI:1.61–2.08; *p* < 0.0001) and intermediate care facility discharge (RR2.71; 95%CI:1.45–5.05; *p* = 0.002). Frail (Hazard Ratio [HR]3.27; 95%CI:1.93–5.55; *p* < 0.0001) and pre-frail patients (HR2.30; 95%CI:1.29–4.09; *p* = 0.005) had worse mid-term mortality (median follow-up 1 years [range 0.5–4 years]). After adjustment for baseline imbalances, frailty was still associated with greater operative mortality (odds ratio [OR]1.97; 95%CI:1.51–2.57; *p* < 0.00001), intermediate care facility discharge (OR4.61; 95%CI:2.78–7.66; *p <* 0.00001) and midterm mortality (HR1.37; 95%CI:1.03–1.83; *p* = 0.03).

**Conclusion:**

In patients undergoing cardiac surgery, frailty and pre-frailty were associated with 2-fold and 1.5-fold greater adjusted operative mortality, respectively, greater adjusted perioperative complications and frailty was associated with almost 5-fold risk of non-home discharge.

**Graphical abstract:**

Burden of frailty and pre-frailty on cardiac surgical outcomes. 
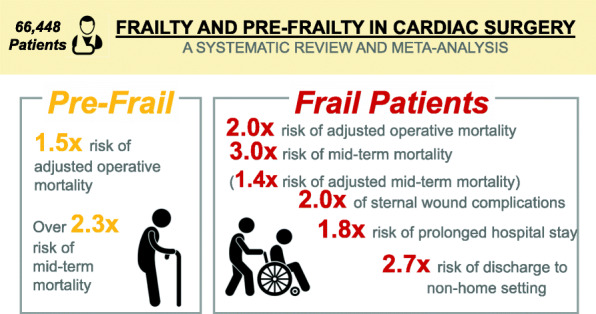

**Supplementary Information:**

The online version contains supplementary material available at 10.1186/s13019-021-01541-8.

## Background

Patients undergoing cardiac surgery are progressively older with greater comorbidities [1]. This can be attributed, in part, to the aging population and to improvements in surgical outcomes in the older adult patient cohort [2, 3]. Frailty, which is commonly found in older adults, can be defined as vulnerability to stressors due to loss of physiologic reserve [4, 5]. Furthermore, Fried et al. [5] reported the intermediate phenotype of “pre-frailty” in roughly half of patients over 65 years. Frailty measures can be broadly conceptualized as phenotypic, including single performance measures and established scores, and index-based tools that consider the accumulation of conditions, signs, symptoms and disabilities [4].

Patients who are deemed frail have been observed to experience higher operative mortality, prolonged intensive care unit (ICU) and hospital length of stay and more frequent discharge to secondary facilities [6]. Despite the growing proportion of frail patients undergoing cardiac surgery, this vulnerable patient population is often excluded from most prospective surgical trials [7, 8]. Few studies have considered the surgical outcomes in the frail and pre-frail population [9]. The objective of this analysis was to better understand the burden of frailty by conducting a systematic review and meta-analysis to compare mortality and secondary patient-centred outcomes of primarily conventional CABG and/or valve procedures in frail, pre-frail and non-frail older adult patients.

## Methods

### Data sources

We systematically searched OVID versions of MEDLINE and EMBASE (1996 to 2018 June week 4 [performed on July 3, 2018]) for studies that mentioned “frail*” and “coronary artery bypass” or “coronar*” or “card*” and “surg*” in the title or abstract (Appendix). This study was performed and reported according to the PRISMA Standard Reporting Guidelines.

### Study selection

Citations were reviewed independently by two reviewers. We included all studies examining adults (no age cut-off) undergoing primarily CABG and valve surgery [8–29]. One study reported on patients undergoing total aortic arch repair [12] and one study included a minority (15%) of transcatheter aortic valve replacements [27]. Pre-frail patients included those classified as “borderline,” [15] “intermediate,” [27] “moderate” [26], “middle tertile” (for 5 m gait speed) [14] and “pre-frail” [9, 20] (Supplementary Table 1). The following reports were considered duplicate studies and analyzed together ([20, 27] [26, 28, 29]).

### Data extraction and quality assessment

Three reviewers independently abstracted data including details of the publication, inclusion/exclusion criteria, patient demographics and cardiac risk factors, description of the interventions used, and outcome definitions and events. Study quality was assessed looking at the following indicators: retrospective versus prospective data collection, concurrent controls, comparable baseline characteristics, completeness of follow-up, and internal consistency of data presented. Disagreements on article inclusion were resolved by consensus.

### Statistical analysis

All analyses were performed using Review Manager (RevMan version 5.2; Cochrane Collaboration, Oxford, UK) and random effects models, which incorporate between-trial heterogeneity and give wider and more conservative confidence intervals (CI) when heterogeneity is present [30]. We assessed statistical heterogeneity among trials using *I*^*2*^, defined as the percentage of total variability across studies attributable to heterogeneity rather than chance, and used published guidelines for low (*I*^*2*^ = 25 to 49%), moderate (*I*^*2*^ = 50 to 74%) and high (*I*^*2*^ ≥ 75%) heterogeneity [31]. For peri-operative outcomes relative risks (RR) was used to pool binary outcomes and mean difference (MD) to pool continuous outcomes. Adjusted binary outcomes were reported as odds ratios (OR). For long-term outcomes OR were used when all patients were followed for the same period of time and hazard ratios (HR) when there was different follow up between groups; these were pooled on the logarithmic scale using the generic inverse variance method. When hazard ratios were not provided, they were approximated as the ratio of the Kaplan-Meier survival curve estimates for each group, and the log-rank survival curve *p*-value was used to estimate the standard error (this method was required to estimate the unadjusted but not adjusted mortality HR for one study [17]). Individual trial and pooled summary results are reported with 95% Cis.

## Results

### Description of included studies

The initial search resulted in 1297 citations from MEDLINE and EMBASE and 78 studies were retrieved for full text review (Supplementary Fig. 1). There were 19 unmatched observational studies with 66,448 patients (15,278 [23%] frail; 6304 [9%] pre-frail; 44,866 [68%] non-frail) that met inclusion criteria. All studies were observational studies with concurrent controls, 6 were multicentre and 13 were single centre. Follow up was to hospital discharge or 30 days in a third (*n* = 7) of the studies, 1 year for all patients in a third (*n* = 6), and variable durations (mean or median follow up of 0.5, 0.75–1, 1.4, 1.8, 4, and 5.4 years) in the remaining third.

### Frailty assessment

Frailty assessment was performed with an established score in 63% and using a single objective test in 37%. The most common frailty scores used were Fried/Modified Fried (25%), Deficit Index (17%), Bespoke Frailty Score (17%), Clinical Frailty Score (8%) and Katz Index (8%). The most common objective tests were walking velocity (71%), 6-min walk test (14%) and psoas muscle measurement (14%).

### Description of included patients

Patients that were classified as being frail were older (MD:+ 2.37; 95%CI:+ 1.30 to + 3.45 years; *p* < 0.0001) and more likely to be female (RR:1.69; 95%CI:1.47–1.94; *p* < 0.00001) than non-frail patients (Supplementary Figs. 2–4). Frail patients had lower hematocrit (MD:-3.36%; 95%CI:-6.59 to − 0.13; *p* = 0.04) and serum albumin (MD:-1.93 g/L; 95%CI:-3.06 to − 0.80; *p* = 0.01; Supplementary Fig. 4). Frail patients also had significantly greater concurrent comorbidities including diabetes (RR:1.35; 95%CI:1.20–1.51; *p* < 0.00001), chronic obstructive pulmonary disease (RR:1.44; 95%CI:1.26–1.64; *p* < 0.00001), previous stroke (RR:2.37; 95%CI:1.99–2.82; *p <* 0.00001), peripheral vascular disease (RR:1.50; 95%CI:1.35–1.66; *p <* 0.00001), chronic kidney disease (RR:1.67; 95%CI:1.44–1.93; *p <* 0.00001), congestive heart failure (RR:1.54; 95%CI:1.25–1.89; *p <* 0.0001), dementia (RR:7.51; 95%CI:1.11–50.61; *p <* 0.0001) and other comorbidities (Supplementary Fig. 3).

Frail patients had higher risk scores including Society of Thoracic Surgery-Predicted Risk of Mortality (STS PROM) (MD:+ 1.38%; 95%CI:+ 0.81 to + 1.94%; *p* < 0.00001), Logistic European System for Cardiac Operative Risk Evaluation (EuroSCORE) (MD:+ 4.93%; 95%CI:+ 0.86 to + 9.00%; *p* = 0.02) and EuroSCORE II (MD:+ 0.97%; 95%CI:+ 0.62 to + 1.32%; *p* < 0.0001) (Supplementary Fig. 4).

### Clinical outcomes

**Fig. 1:**
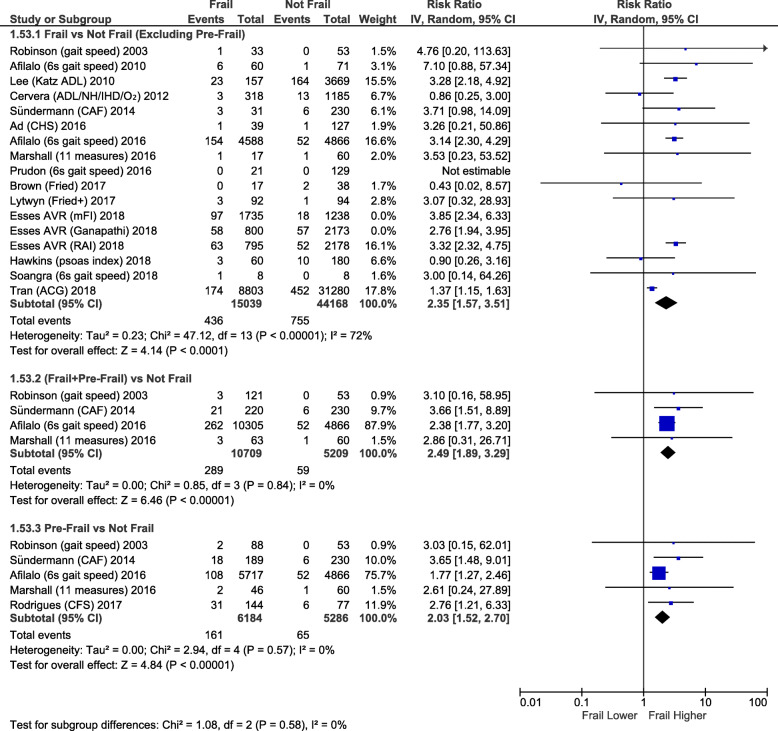
Forest Plot for unadjusted operative mortality. Individual study and pooled unadjusted risk ratios (RRs) of frail vs non-frail patients undergoing primarily CABG and valve surgery. The pooled RR and OR with 95% CI were calculated using random-effects models. Esses et al. [22] provided (unadjusted) results using three different frailty indices: modified frailty index (mFI), Ganapathi index, and risk analysis index (RAI). All three results are shown in the unadjusted figure but only the RAI results were used to calculate the pooled results in the figure. The pooled results are similar if the modified frailty index (RR 2.38, 95% CI 1.57 to 3.62, *p* < 0.0001; *I*^*2*^ = 72%) or Ganapathi index (RR2.28, 95%CI:1.56–3.35, *p* < 0.0001; *I*^*2*^ = 69%) are used. *Sensitivity analyses – Unadjusted risk of operative mortality also higher if the two studies with largest weighting (*[14, 24]*) are excluded: RR2.99, 95%CI:2.34–3.82, p < 0.00001, I*^*2*^ *= 0% (the unadjusted risk of operative mortality remains higher if the next two largest weightings are also excluded (*[6, 22]*): RR1.85, 95%CI:1.02–3.34, p = 0.04, I*^*2*^ *= 0%)*

**Fig. 2:**
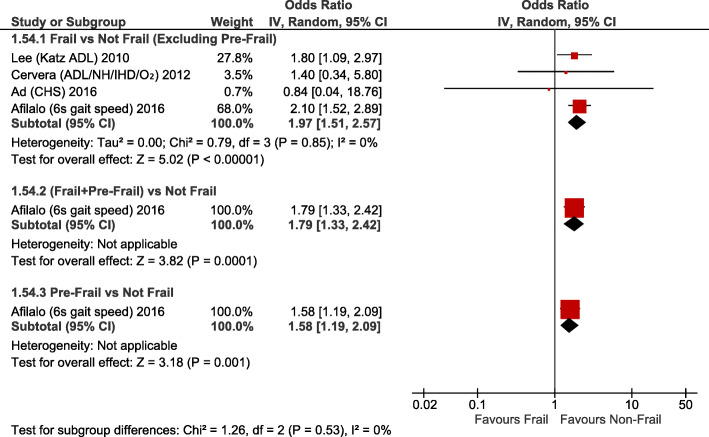
Forest Plot for adjusted operative mortality. Individual study and pooled adjusted odds ratios (ORs) of frail vs non-frail patients undergoing primarily CABG and valve surgery. The pooled RR and OR with 95% CI were calculated using random-effects models. *Sensitivity analyses – Adjusted risk of operative mortality is also higher if study with largest weighting* [14] *is excluded: adjusted OR 1.72, 95% CI 1.08–2.75, p = 0.02, I*^*2*^ *= 0%*

**Fig. 3:**
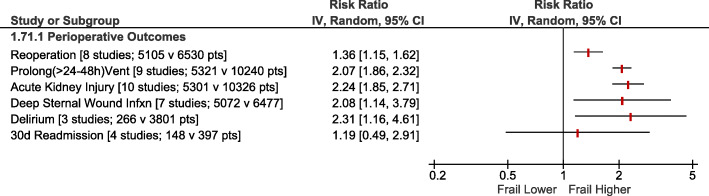
Forest Plot for perioperative complications. Individual study and pooled unadjusted risk ratios (RRs) of frail vs non-frail patients undergoing primarily CABG and valve surgery. The pooled RRs with 95% CI were calculated using random-effects models. *Sensitivity Analyses – Removing the results of the study with largest weighting* [14] *made the pooled results for reoperation [90% weighting, RR1.57, 95%CI:0.90–2.71, p = 0.11, I*^*2*^ *= 0%] and deep sternal wound infection [56% weighting, RR1.29, 95%CI:0.52–3.17, p = 0.58, I*^*2*^ *= 0%] no longer statistically significant, but the results for prolonged ventilation [57% weighting, RR1.96, 95%CI:1.63–2.36, p < 0.00001, I*^*2*^ *= 5%] and acute kidney injury [51% weighting, RR2.40, 95%CI:1.83–3.15, p < 0.00001, I*^*2*^ *= 0%] remained statistically significantly higher*

**Fig. 4:**
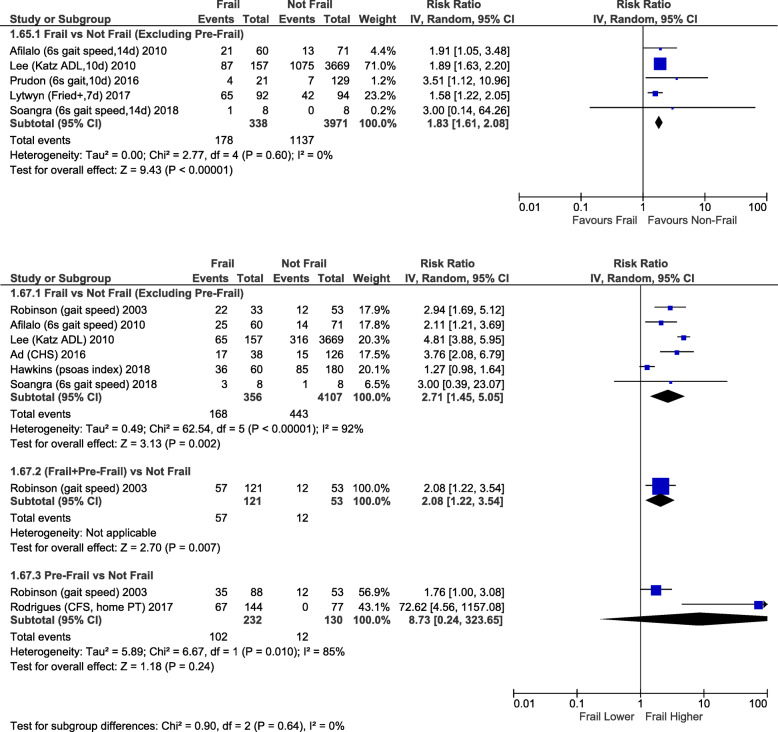
Forest Plot for prolonged hospitalization (Top) and discharge to an intermediate care facility (Bottom). Individual study and pooled unadjusted risk ratios (RRs) of frail vs non-frail patients undergoing primarily CABG and valve surgery. The pooled RRs with 95% CI were calculated using random-effects models. *Sensitivity Analyses – Risk of prolonged hospital stay also higher if study with largest weighting is excluded* [6]*: RR1.69, 95%CI:1.34–2.13, p < 0.0001, I*^*2*^ *= 0%. Adjusted risk of discharge to intermediate care facility is also higher if study with largest weighting is excluded* [6]*: adj OR 3.16, 95%CI:1.66–6.02, p = 0.0005, I*^*2*^ *= 0%*

**Fig. 5:**
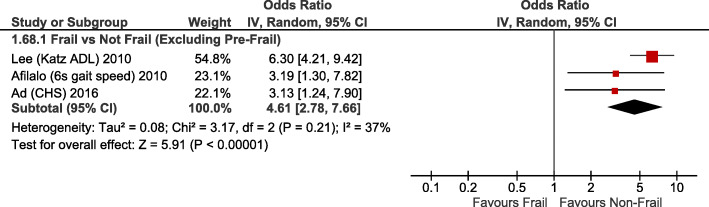
Forest Plot for adjusted discharge to an intermediate care facility. Individual study and pooled adjusted odds ratios (ORs) of frail vs non-frail patients undergoing primarily CABG and valve surgery. The pooled ORs with 95% CI were calculated using random-effects models. *Sensitivity Analyses – Adjusted risk of discharge to intermediate care facility is also higher if study with largest weighting* [6] *is excluded: adjusted OR 3.16, 95% CI 1.66–6.02, p = 0.0005, I*^*2*^ *= 0%*

Frailty (RR2.35; 95%CI:1.57–3.51; *p <* 0.0001) and pre-frailty (RR2.03; 95%CI:1.52–2.70; *p* < 0.00001) were associated with increased operative mortality compared with non-frail patients (Fig. 1). After adjusting for baseline differences, this remained true with greater operative mortality for frailty (adjusted OR1.97; 95%CI:1.51–2.57; *p <* 0.00001) and pre-frailty (adjusted OR1.58; 95%CI:1.19–2.09; *p* = 0.001; Fig. 2). Frailty was also associated with greater risk of perioperative stroke (RR1.36; 95%CI:1.04–1.77; *p* = 0.02; Supplementary Fig. 5) and sternal wound complications (RR2.08; 95%CI:1.14–3.79; p = 0.02; Fig. 3). Frail patients experienced longer ICU length of stay (MD:+ 0.65 days; 95%CI:+ 0.03 to + 1.27; *p* = 0.04; Supplementary Fig. 4), prolonged mechanical ventilation (RR:2.07; 95%CI:1.86–2.32; *p <* 0.00001); longer (MD:+ 1.92 days; 95%CI:+ 1.09 to + 2.75; *p <* 0.00001; Supplementary Fig. 4) and higher risk of prolonged (> 7-14d [definition varied between studies]) (RR1.83; 95%CI:1.61–2.08; *p <* 0.00001; Fig. 4) hospital length of stay; and higher risk for discharge to an intermediate care facility (RR2.71; 95%CI:1.45–5.05; *p* = 0.002; Fig. 4). Frailty was not associated with any difference in 30-day hospital readmission (RR1.19; 95%CI:0.49–2.91; *p* = 0.7; Fig. 3). Discharge to an intermediate facility remained significant following adjustment for baseline differences in frail versus non-frail patients (adjusted OR:4.61; 95%CI:2.78–7.66; *p <* 0.00001; Fig. 5).

**Fig. 6:**
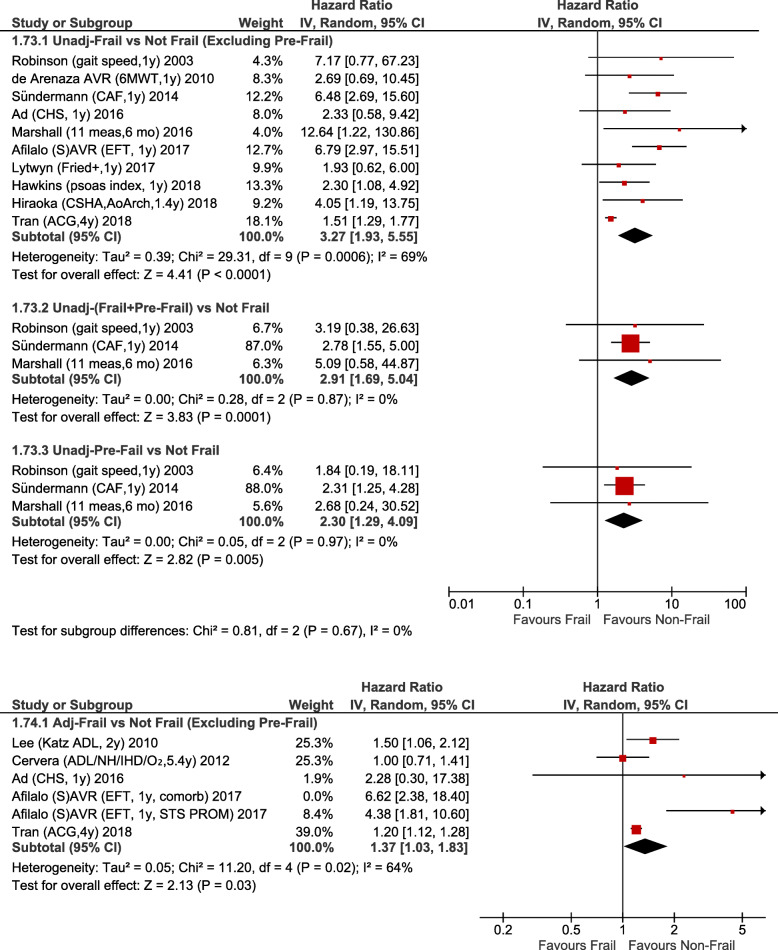
Forest Plot for mid-term mortality. Individual study and pooled unadjusted (Top) and adjusted (Bottom) hazard ratios (HRs) of frail vs non-frail patients undergoing primarily CABG and valve surgery. The pooled HRs with 95% CI were calculated using random-effects models. Afilalo et al. [21] provided two separate adjusted results; the adjusted results using the Society of Thoracic Predicted Risk of Mortality (STS PROM) were used to calculate the pooled adjusted results in the figure. If the other adjusted results using comorbidities were used (also shown in the figure) the pooled adjusted results were similar: HR1.41, 95%CI:1.02–1.96, *p* = 0.04; *I*^*2*^ = 71%. *Sensitivity analyses – Risk of unadjusted long-term mortality remains higher if study with largest weighting is excluded* [24]*: HR3.85, 95%CI:2.63–5.64, p < 0.00001, I*^*2*^ *= 5%) but adjusted mid-term mortality was no longer statistically significant if study with largest weighting is excluded* [24]*: HR1.65, 95%CI:0.95–2.85, p = 0.07, I*^*2*^ *= 71%*

Among the 11 studies that reported unadjusted mid-term mortality, 8 studies continued to follow all patients to 1 year, whereas the other three followed all patients to 6 months, or to an average of 1.4 and 4 years, respectively. Mid-term mortality (median follow-up 1 years [range 0.5–4 years]) was higher for both frail (HR 3.27; 95%CI:1.93–5.55; *p <* 0.0001) and pre-frail patients (HR 2.30; 95%CI:1.29–4.08; *p* = 0.005) (Fig. 6). There was also greater adjusted mid-term mortality for frail versus non-frail patients (adjusted HR 1.37; 95%CI:1.03–1.83; *p* = 0.03) reported by a smaller group of five studies (Fig. 6).

Sensitivity analyses were performed by removing the results of one or more of the largest studies [6, 14, 24] (see also figure captions). Risks remained higher for preoperative patient characteristics, and major outcomes including operative mortality (RR2.99, 95%CI:2.34–3.82, *p <* 0.00001) excluding [14, 24], stroke (RR1.97, 95%CI:1.17–3.30, *p* = 0.01) excluding [14], prolonged ventilation (RR 1.96, 95%CI 1.63–2.36, *p <* 0.00001) excluding [14], acute kidney injury (RR2.40, 95%CI:1.83–3.15, *p <* 0.00001) excluding [14], and prolonged hospital stay (RR1.69, 95%CI:1.34–2.13, *p <* 0.0001) excluding [6]. The pooled results for reoperation (RR 1.57, 95%CI:0.90–2.71, *p* = 0.11) and deep sternal wound infection (RR1.29, 95%CI:0.52–3.17, *p* = 0.58) were no longer statistically significant after excluding [14]. For perioperative outcomes adjusted for baseline differences, risks remained higher for operative mortality (adjusted OR1.72, 95%CI:1.08–2.75, *p* = 0.02) excluding [14], and discharge to intermediate care facility (adjusted OR3.16, 95%CI:1.66–6.02, *p* = 0.0005) excluding [6]. For long-term mortality, unadjusted risk remains higher (HR3.85, 95%CI:2.63–5.64, *p <* 0.00001) excluding [24], but adjusted risk no longer statistically significant (adjusted HR1.65, 95%CI:0.95–2.85, *p* = 0.07) excluding [24].

To address the variability in the frailty measures used among studies, we analyzed the only 4 studies that used the same frailty measure [10, 14, 18, 19], separately as a subset as an additional sensitivity analysis (Supplemental Table 2). The outcomes of this subset are generally similar to the pooled results of all the studies, though some of the results for the subset do not achieve statistical significance due to the reduced numbers of studies and patients.

## Discussion

To our knowledge, this is one of the first systematic reviews and meta-analysis comparing the outcomes of frail, pre-frail and non-frail patients undergoing cardiac surgery. A strength of this review is that it systematically summarizes all the published data in this field with the inclusion of a large number studies. We found that 1) a range of frailty scores and objective measures were used to assess frailty; 2) frail patients were older, more likely to be female and had greater co-morbidities; 3) frailty as well as pre-frailty were associated with greater operative mortality and decreased long term survival post-cardiac surgery, even after adjusting for differences in baseline risk; 4) frailty was associated with greater risk of stroke, sternal wound complications, extended stay in hospital and discharge to an intermediate care facility.

Despite improvements in surgical outcomes, frailty still portends an almost doubling in adjusted operative mortality. This is of particular relevance to patients, their caregiver and their healthcare providers in the timing and potentially the type of surgical intervention including the decision to not undergo surgery. For such patients, one may consider less invasive transcatheter procedures for coronary or valvular disease. Alternatively, preoperative rehabilitation or “prehab” – a set of interventions to improve patient mental, nutritional status and/or physical capacity to “defrail” elective patients – may be considered [32]. This is a burgeoning field and the Pre-operative Rehabilitation for Reduction of Hospitalization After Coronary Bypass and Valvular Surgery is an ongoing multicenter, randomized controlled trial with an aim to examine the effect of prehab consisting of 8-weeks of exercise and education intervention in frail patients (https://clinicaltrials.gov/ct2/show/NCT02219815).

As mentioned, there is greater appreciation for the importance of frailty in patients undergoing cardiac surgery but the optimal method of assessment beyond the “eye-ball” test remains unknown [4]. The ideal measurement would be fast, easy, reproducible and a strong independent risk predictor for cardiac surgical outcomes. Afilalo and colleagues [14] demonstrated that patients with a slow gait speed had a 2- to 3-fold increased risk of mortality beyond the STS PROM and EuroSCORE. In the 2011 revision of the Adult Cardiac Surgery Database, the STS incorporated the measurement of gait speed to assist clinicians in the identification of cardiac surgery patients who are at increased risk for adverse outcomes. Another study by Afilalo and colleagues [21] comparing several commonly-used measures demonstrated variability in the proportion of patients classified as frail, however differences in unadjusted and adjusted mortality exhibited low overall heterogeneity (*I*^*2*^ = 0–22%) among the different measures (Supplementary Fig. 6).

Frailty was associated with greater risk of almost all cardiac surgical complications, resulting in extended ICU and hospital stay as well as discharge to a non-home setting. Even pre-frailty, found in roughly half of older cardiac patients, was associated with a significant risk of adverse post-operative outcomes. Thus, the care of older frail patients carries a significant and disproportionate burden of ICU and hospital and overall healthcare resources [33]. Unfortunately, there was insufficient data in the included studies to perform a proper cost analysis.

## Limitations

There are several limitations that deserve mention. All studies were observational with few propensity-adjusted analyses, which is important given the large number of baseline differences between frail and non-frail patients. Frailty is a difficult syndrome to quantitatively define as there is a yet a lack of universally accepted definition of its presence [34]. We attempted to address this variability by analyzing the only 4 studies that used the same frailty measure separately as a subset. As such, there was a range of tools and measures in the included studies. We used established cut-offs and groupings to form categories of frail, pre-frail and non-frail. These different classifications likely resulted in intermediate-phenotype patients either being reported with either the frail or non-frail patient groups in different studies. It was not possible to determine whether operative or midterm mortality was cardiac-related, and variable follow up between studies is a further limitation in pooling midterm outcomes. Finally, we were unable to capture the time taken for full recovery to a baseline quality of life post-sternotomy, which can be protracted in the frail patient cohort.

## Conclusions

Based on the results of this meta-analysis, frailty was associated with an almost 2-fold greater operative mortality and an almost 1.5 fold greater medium term mortality, even after adjusting for differences in baseline risk. Frailty was associated with a range of perioperative complications as well as 5-fold risk of discharge to an intermediate care facility. Frailty as well as pre-frailty were associated with a 2-fold and 1.5-fold decease in mid-term survival even after adjusting for differences in baseline risk, respectively. Our analysis supports the routine assessment of frailty in patients who are being evaluated to undergoing a surgical intervention. The utility of this data may provide a signal for a measured approach to cardiac surgery in the frail patient cohort and lends credence to the on-going study of prehab.

### Supplementary Information

**Additional file 1: Supplementary Fig. 1**: MEDLINE and EMBASE were searched for all records until July 2018. Abstracts were reviewed for 1297 citations. 78 studies were retrieved for full text review and 19 studies met inclusion criteria following full article review. **Supplementary Fig. 2**: Forest Plot for age in frail vs non-frail patients undergoing primarily CABG and valve surgery. The pooled mean difference (MD) with 95% CI was calculated using random-effects models. To include Marshall et al. [15], which provided means but not standard deviations for age, we imputed the largest standard deviation from the other studies. Alternatively, excluding [15] does not significantly change the pooled result: MD + 2.33, 95%CI:+ 1.25 to + 3.41 years for Frail vs Not Frail, MD + 2.66, 95%CI:+ 1.85 to + 3.48 years for Frail+Pre-Frail vs Not Frail, and MD + 2.00, 95%CI:+ 1.74 to + 2.25 years for Pre-Frail vs Not Frail subgroups. **Supplementary Fig. 3**: Forest Plot for baseline and operative characteristics in frail vs non-frail patients undergoing primarily CABG and valve surgery (binary outcomes). The pooled risk ratios (RRs) with 95% CI were calculated using random-effects models. **Supplementary Fig. 4**: Forest Plot for baseline and operative characteristics in frail vs non-frail patients undergoing primarily CABG and valve surgery (continuous outcomes). The pooled mean differences (MDs) with 95% CI were calculated using random-effects models. To include Marshall et al. [15], which provided means but not standard deviations for age, log EuroSCORE, and EuroSCORE II, we imputed the largest standard deviation from the other studies. Alternatively, excluding [15] does not significantly change the pooled results: 1) Age – MD + 2.33, 95%CI:+ 1.25 to + 3.41, *p* < 0.0001, 14 studies, 14,321 v 41,901 patients; 2) log EuroSCORE (%) – MD + 3.68, 95%CI:–0.27 to + 7.62, *p* = 0.07, 3 studies, 112 v 430 patients; and 3) EuroSCORE II (%) – MD + 0.96, 95%CI:+ 0.61 to + 1.31, *p* < 0.00001, 3 studies, 152 v 350 patients. **Supplementary Fig. 5**: Forest Plot for stroke. Individual study and pooled unadjusted risk ratios (RRs) of frail vs non-frail patients undergoing primarily CABG and valve surgery. The pooled RRs with 95% CI were calculated using random-effects models. *Sensitivity analysis – Risk of stroke higher if the study with the largest weighting is excluded* [14]*: RR 1.97, 95% CI 1.17–3.30, p = 0.01, I*^*2*^ *= 0%.* **Supplementary Fig. 6**: Forest Plot showing differences in 1 year mortality results for each of the 6 different frailty measures used in Afilalo et al. [21]: unadjusted 1 year mortality (Top), 1 year mortality adjusted for comorbidities (Middle) and 1 year mortality adjusted for Society of Thoracic Surgery Predicted Risk of Mortality (STS PROM) (Bottom).

**Additional file 2 Supplementary Table 1**: Characteristics of included studies. **Supplementary Table 2**: Comparisons of Pooled Outcomes Using All Studies vs Only Studies Using the Same 5 Metre/6 Second Walk Test Frailty Measure

## Data Availability

All data generated or analyzed during this study are included in this published article and its supplementary information files.

## Appendix

### Appendix 1: Search Strategy

Database: Ovid MEDLINE: Epub Ahead of Print, In-Process & Other Non-Indexed Citations, Ovid MEDLINE® Daily and Ovid MEDLINE® < 1946–2018 June Week 4>, Embase Classic+Embase < 1947 to 2018 July 03>.

Search Strategy:

-----------------------------------------------------------------

1 frail*.mp. (48772).

2 coronary artery bypass.mp. or exp. coronary artery bypass graft/ (152566).

3 card*.mp. (3494244).

4 coronar*.mp. (1146987).

5 surg*.mp. (6804471).

6 1 and (2 or ((3 or 4) and 5)) (1722).

7 remove duplicates from 6 (1297).

## Abbreviations

AVR: Aortic valve replacement
CABG: Coronary artery bypass graft
EuroSCORE: European System for Cardiac Operative Risk Evaluation
NYHA: New York Heart Association
STS PROM: Society of Thoracic Surgeons Predicted Risk of Mortality
