# Supplementary material for: Frailty and pre-frailty in cardiac surgery: a systematic review and meta-analysis of 66,448 patients
Source: J Cardiothorac Surg. 2021 Jun 25;16:184. doi: 10.1186/s13019-021-01541-8 (PMC8229742; doi:10.1186/s13019-021-01541-8)
Supplement: Supplementary file 1 — Additional file 1: Supplementary Fig. 1: MEDLINE and EMBASE were searched for all records until July 2018. Abstracts were reviewed for 1297 citations. 78 studies were retrieved for full text review and 19 studies met inclusion criteria following full article review. Supplementary Fig. 2: Forest Plot for age in frail vs non-frail patients undergoing primarily CABG and valve surgery. The pooled mean difference (MD) with 95% CI was calculated using random-effects models. To include Marshall et al. [15], which provided means but not standard deviations for age, we imputed the largest standard deviation from the other studies. Alternatively, excluding [15] does not significantly change the pooled result: MD + 2.33, 95%CI:+ 1.25 to + 3.41 years for Frail vs Not Frail, MD + 2.66, 95%CI:+ 1.85 to + 3.48 years for Frail+Pre-Frail vs Not Frail, and MD + 2.00, 95%CI:+ 1.74 to + 2.25 years for Pre-Frail vs Not Frail subgroups. Supplementary Fig. 3: Forest Plot for baseline and operative characteristics in frail vs non-frail patients undergoing primarily CABG and valve surgery (binary outcomes). The pooled risk ratios (RRs) with 95% CI were calculated using random-effects models. Supplementary Fig. 4: Forest Plot for baseline and operative characteristics in frail vs non-frail patients undergoing primarily CABG and valve surgery (continuous outcomes). The pooled mean differences (MDs) with 95% CI were calculated using random-effects models. To include Marshall et al. [15], which provided means but not standard deviations for age, log EuroSCORE, and EuroSCORE II, we imputed the largest standard deviation from the other studies. Alternatively, excluding [15] does not significantly change the pooled results: 1) Age – MD + 2.33, 95%CI:+ 1.25 to + 3.41, p < 0.0001, 14 studies, 14,321 v 41,901 patients; 2) log EuroSCORE (%) – MD + 3.68, 95%CI:–0.27 to + 7.62, p = 0.07, 3 studies, 112 v 430 patients; and 3) EuroSCORE II (%) – MD + 0.96, 95%CI:+ 0.61 to + 1.31, p < 0.00001, 3 studies, 1 [file 13019_2021_1541_MOESM1_ESM.pptx]

## Slide 1
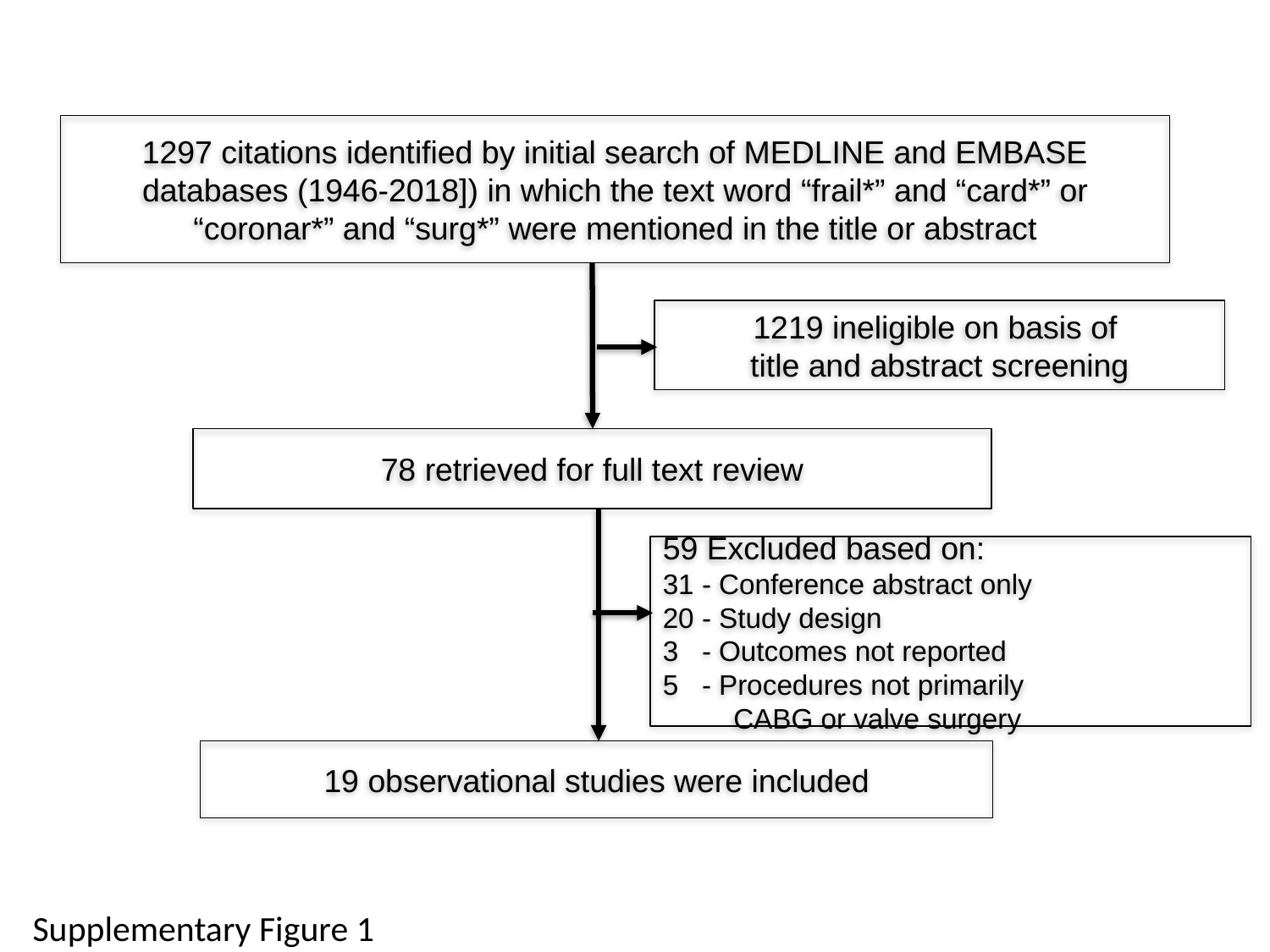

1297 citations identified by initial search of MEDLINE and EMBASE databases (1946-2018]) in which the text word “frail*” and “card*” or “coronar*” and “surg*” were mentioned in the title or abstract
1219 ineligible on basis of
title and abstract screening
78 retrieved for full text review
59 Excluded based on:
31 - Conference abstract only
20 - Study design
3 - Outcomes not reported
5 - Procedures not primarily
 CABG or valve surgery
19 observational studies were included
Supplementary Figure 1

## Slide 2
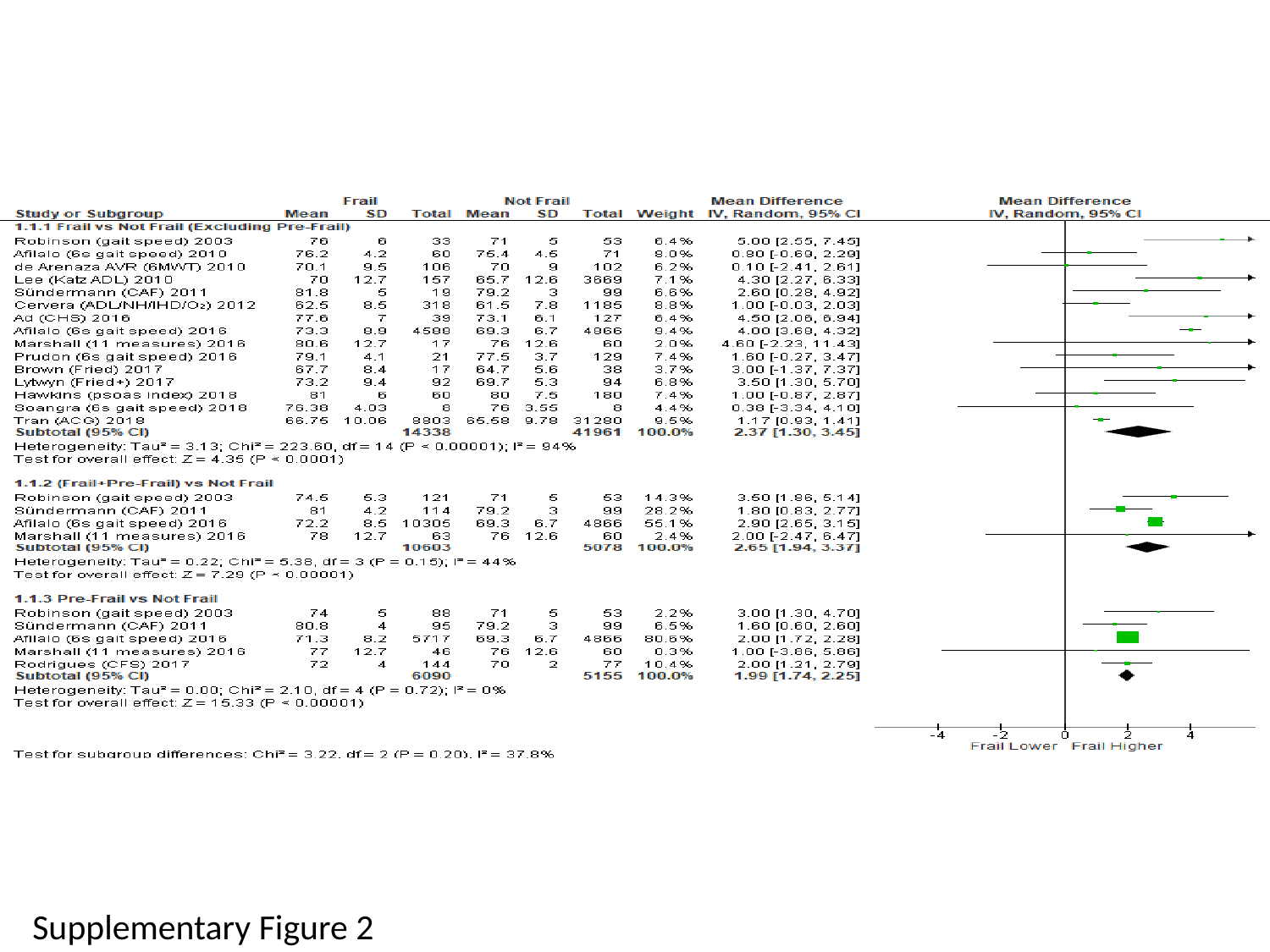

Supplementary Figure 2

## Slide 3
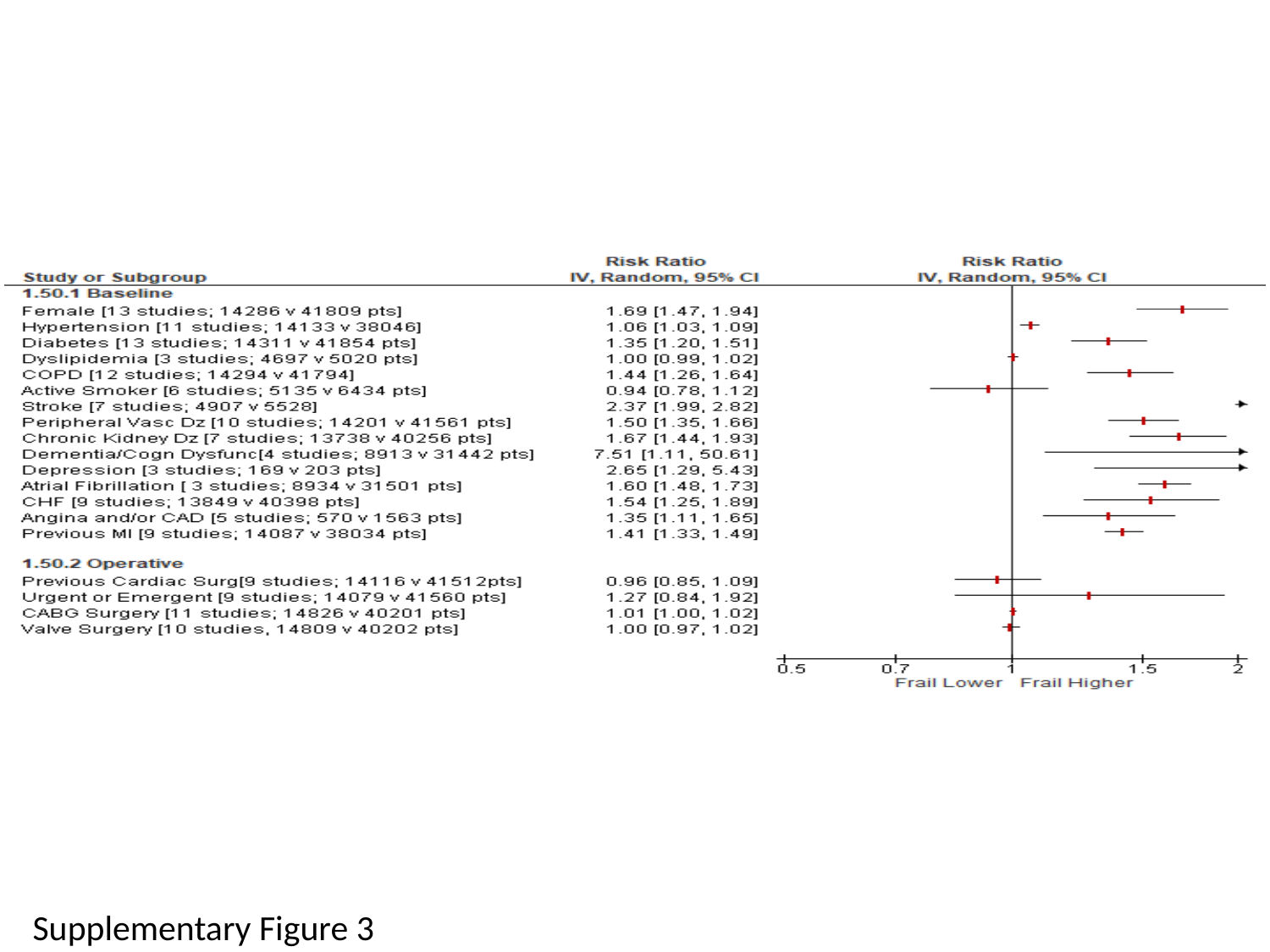

Supplementary Figure 3

## Slide 4
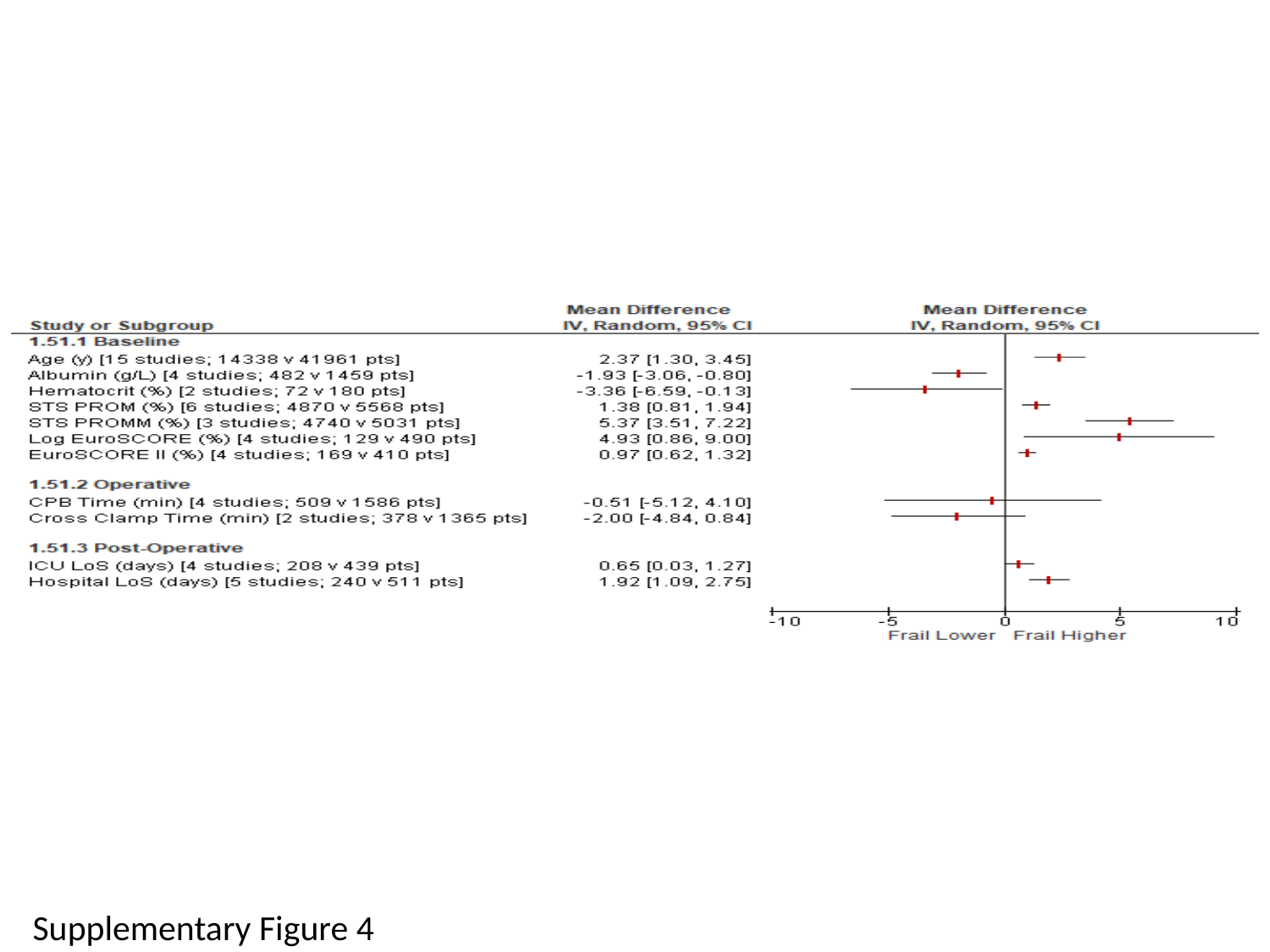

Supplementary Figure 4

## Slide 5
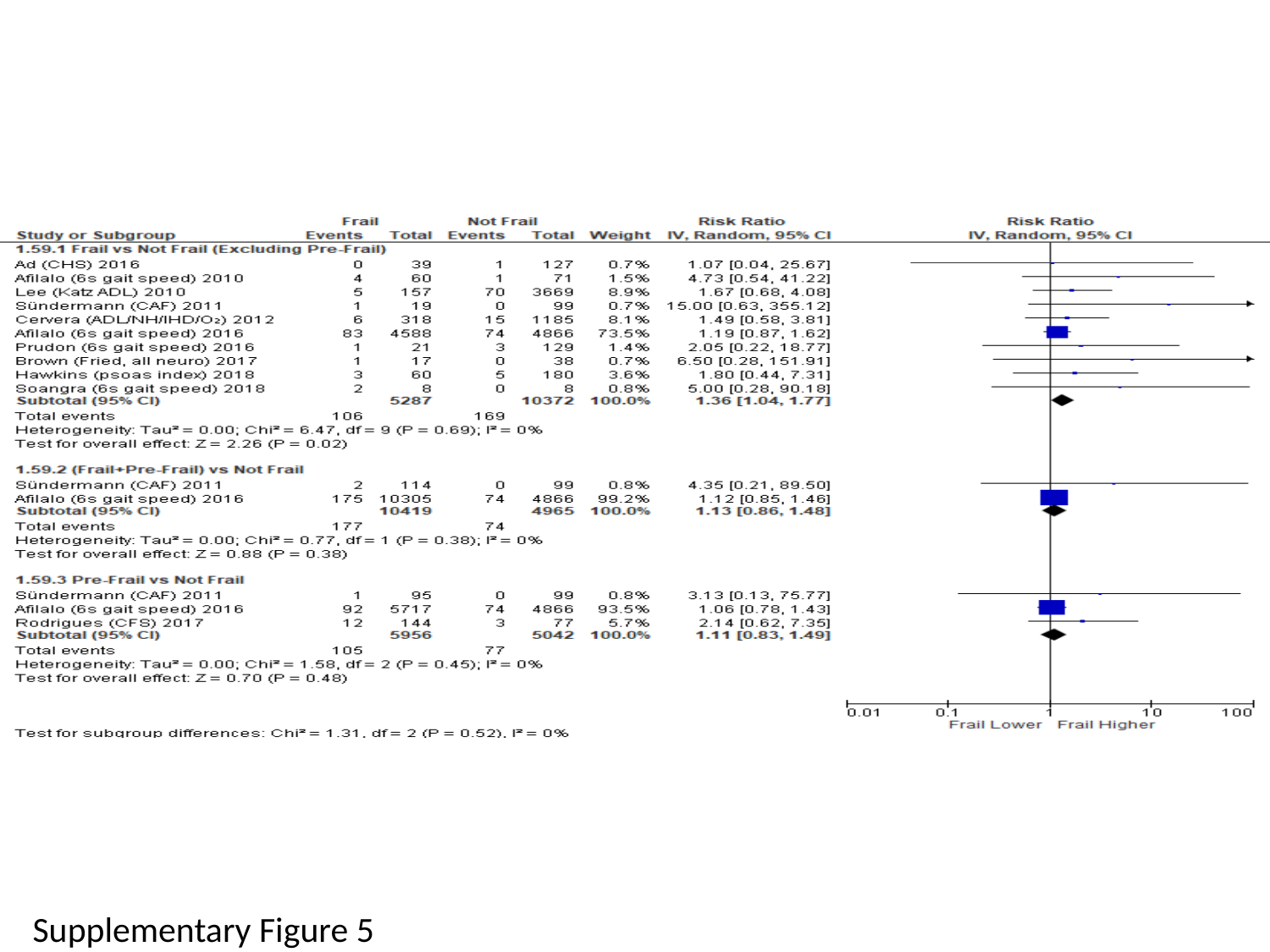

Supplementary Figure 5

## Slide 6
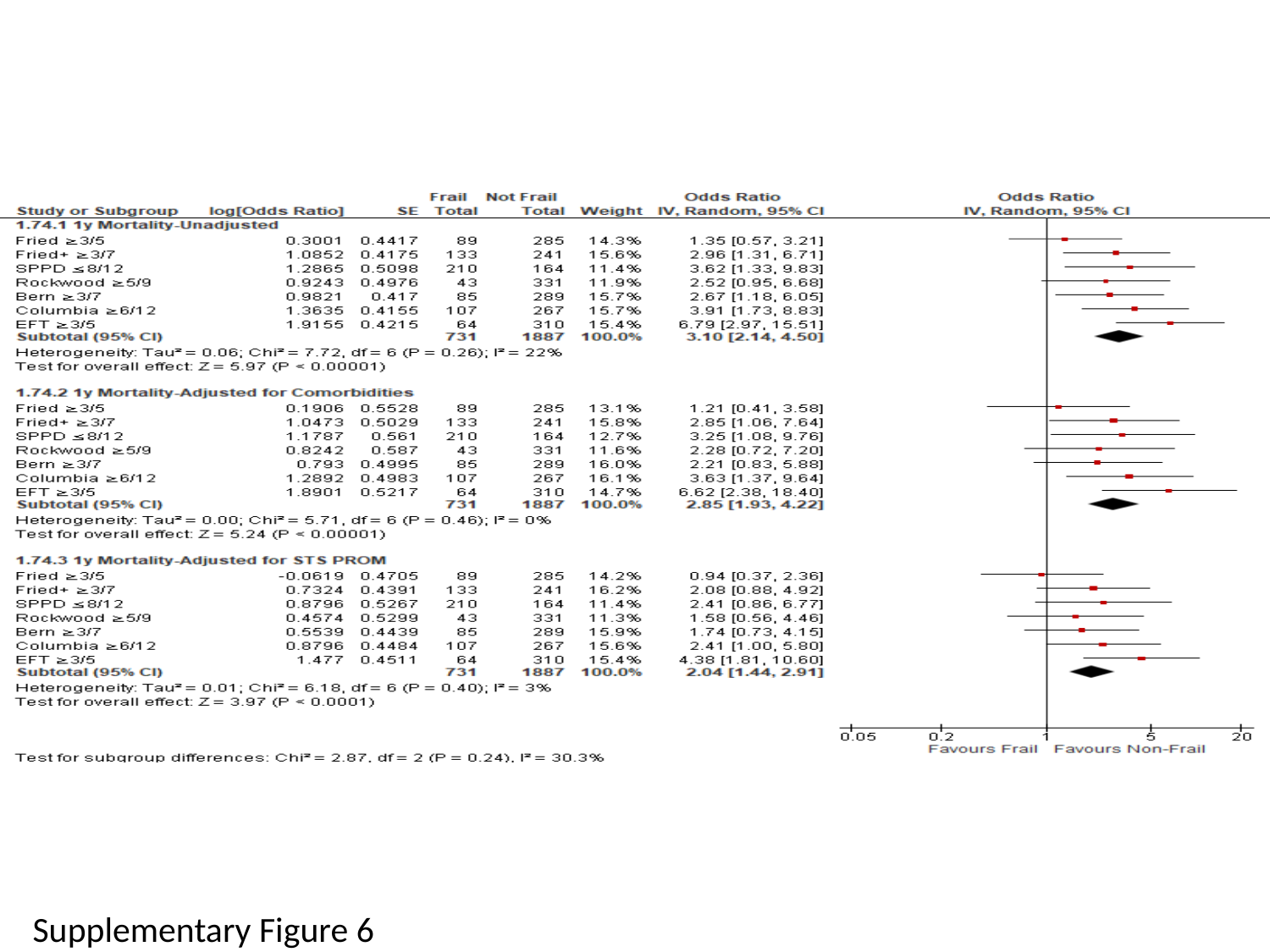

Supplementary Figure 6
