# Supplementary material for: Frailty and pre-frailty in cardiac surgery: a systematic review and meta-analysis of 66,448 patients
Source: J Cardiothorac Surg. 2021 Jun 25;16:184. doi: 10.1186/s13019-021-01541-8 (PMC8229742; doi:10.1186/s13019-021-01541-8)
Supplement: Supplementary file 2 — Additional file 2 Supplementary Table 1: Characteristics of included studies. Supplementary Table 2: Comparisons of Pooled Outcomes Using All Studies vs Only Studies Using the Same 5 Metre/6 Second Walk Test Frailty Measure [file 13019_2021_1541_MOESM2_ESM.docx]

**Supplementary Table 1**: Characteristics of included studies

| Author  Year Country  Enrolment Period (yr) | No. of Centers | No. of Patients | Follow Up | Prospective/ Retrospective (inclusion and exclusion criteria) | Frailty Measure and  Comparable Baseline |
| --- | --- | --- | --- | --- | --- |
| Lee 2009 [6]  Canada  (2004-2007) | 1 | N=3826; 157 Frail vs 3669 non-frail | 1.8 (0.9 to 2.8) years [median (IQR)] | Prospective | Frail: Deficiency in either the Katz index of activities of daily living, independent ambulation or dementia  Non-frail: None of the above |
| Afilalo 2010 [19]  USA and Canada  (2008-2009) | 4 | N=131; 60 Frail vs 71 non-frail | Hospital length of stay [all] | Prospective (age 70+, elective valve or CABG cases only) | Frail: Gait speed test 6 or more seconds for 5 m (≤ 0.83 m/s)  Non-frail: Gait speed test less than 6 seconds for 5 m (> 0.83 m/s) |
| de Arenaza 2010 [23]  UK, Poland, Belgium, Norway  (prior to 2005) | 10 | N= 208; 106 Frail vs 102 non-frail | 1 year [median] | Prospective Randomized (secondary analysis) (AVR cases only) | Frail: 6 minute walk test < 300m  Non-frail: 6 minute walk test ≥300m |
| Cervera 2012 [25]  USA  (1997-2009) | 1 | N=1508; 318 Frail vs 1185 non-frail | 5.4 (0.1-13.1) years [median (range)] | Retrospective (isolated CABG cases only) | Frail: Functional impairment (needing device for ADLs, from nursing home, on dialysis or oxygen therapy)  Non-frail: Without functional impairment |
| Robinson 2013 [20]  USA  (2007-2010) | 1 | N=129 (cardiac operations only) ; 32 Frail vs 25 pre-frail vs 72 non-frail | 30 days or entire hospital length of stay [all] | Prospective (age 65+, elective cases only) | Frail: Bespoke frailty score 4-7  Pre-frail: Bespoke frailty score 2-3  Non-frail: Bespoke frailty score 0-1 |
| Robinson 2013 [27]  USA  (2007-2011) | 1 | N=174 (cardiac operations only); 33 Frail vs 88 pre-frail vs 53 non-frail | Minimum 1 year follow up [all] | Prospective (age 65+, elective cases only) | Frail: Time up and go test (10 feet) – 15 or more seconds  Pre-frail: Time up and go test (10 feet) – 11-14 seconds  Non-frail: Time up and go test (10 feet) – 10 or less seconds  (Stand from chair, walk 10 feet then sit down) |
| Sündermann 2014 [26] Germany (2008-2010) | 1 | N=450; 31 (severely) frail vs 189 (moderately) frail vs 230 non-frail | 1 year [all] | Prospective (age 74+, elective cases only; 15% underwent TAVI) | Severely frail: Comprehensive Assessment of Frailty test (CAF) score 1-10  Moderately (pre-)frail: CAF score 11-25  Non-frail: CAF score 26-35 |
| Ad 2016 [17]  USA (2012-2013) | 1 | N=166; 39 Frail vs 127 non-frail | 30 days [all] | Prospective (age 65+, elective cases only) | Frail: Cardiovascular Health Study (CHS) score 3 or more  Non-frail: CHS score 2 or less |
| Afilalo 2016 [14]  USA  (2011-2014) | 109 | N=15171; 4588 Frail vs 5717 pre-frail vs 4866 non-frail | 30 days [all] | Retrospective (age 60+, CABG, valve or CABG + valve surgery cases) | Frail: Walking velocity < 0.83 m/s in 5 m walk test  Pre-frail: Walking velocity 0.83-1 m/s in 5 m walk test  Non-frail: Walking velocity >1 m/s in 5 m walk test |
| Brown 2016 [16]  USA  (2010-2013) | 1 | N=55; 17 Frail vs 38 non-frail | 30 days [all] | Prospective (age >55, isolated elective first-time CABG only) | Frail: Fried frail score 3 or more  Non-frail: Fried frail score 2 or lower |
| Marshall 2016 [15]  Australia  (2012-2013) | 1 | N=123; 17 Frail vs 46 pre-frail vs 60 non-frail | 6 months [all] | Prospective (age 70+, elective or urgent inpatients) | Frail: Bespoke frailty score 4+  Pre-frail: Bespoke frailty score 2-3  Non-frail: Bespoke frailty score 0-1 |
| Prudon 2016 [18]  Netherlands  (2012-2013) | 1 | N=150; 21 Frail vs 129 non-frail | 30 days or entire hospital length of stay [all] | Prospective (age 70+, elective valve or CABG +/- other) | Frail: Gait speed test 6 or more seconds to walk 5 m (≤0.83 m/s)  Non-frail: Gait speed test less than 6 seconds to walk 5 m (>0.83 m/s) |
| Afilalo 2017 [21]  USA, Canada, France  2012-2015 | 14 | N=374 (SAVR only); 64 frail vs 310 non-frail | 1 year [all] | Prospective (age 70+ undergoing TAVR or SAVR ±CABG) | Frail: Essential Frailty Toolset (EFT) score 3-5  Non-frail: EFT score 0-2 |
| Lytwyn 2017 [13]  Canada  (not reported) | 1 | N=186; 92 Frail vs 94 non-frail | 1 year [all] | Prospective (elective or urgent patients) | Frail: Modified Fried frail score 3 or higher  Non-frail: Modified Fried frail score 2 or lower |
| Rodrigues 2017 [9]  Brazil (not reported) | 1 | N=221; 144 Pre-frail vs vs 77 non-frail | 60 days [all] | Prospective (age >65) | Pre-Frail: Clinical Frailty Score (CFS) 4  Non-frail: CFS 1-3  (Frail: CFS 5+ excluded) |
| Esses 2018 [22]  USA  (2006-2012) | ACS-NSQIP database | N=3088; 800 Frail vs 2288 non-frail | 30 days [all] | Retrospective (age 65+ and elective AVRs; excluded concomitant AVR + CABG, ASA 4, and emergency surgery) | Frail: modified frailty index 0.2 or greater, risk analysis index 15 or greater, Ganapathi index 2 or greater  Non-frail: modified frailty index less than 0.2, risk analysis index less than 15, Ganapathi index less than 2 |
| Hawkins 2018 [11]  USA  (2009-2016) | 1 | N=240; 60 Frail vs 180 non-frail | 1 year [all] | Retrospective (first time AVR, STS risk over 3%; excluded endocarditis) | Frail: Psoas index of <6.96 for women and <9.09 for men  Non-frail: psoas index of >6.96 for women and >9.09 for men  (the cutoff was determined at the 25^th^ percentile as evaluated by CT scan) |
| Hiraoka 2018 [12] Japan  (2012-2017) | 1 | N=113; 17 Frail vs 96 non-frail | 1.4 +/- 1.1 years [mean ± sd] | Retrospective (total aortic arch replacement) | Frail: Bespoke frailty score of 6-9 points determined by a combination of age, CSHA scale, serum albumin, Katz index of ADL  Non-frail: Bespoke frailty score of 0-5 points |
| Soangra 2018 [10]  USA  (not reported) | 1 | N=16; 8 Frail vs 8 non-frail | hospital discharge [all] | Prospective (age >70) | Frail: Walking velocity ≤ 0.83 m/s in 5 meter walk test  Non-frail: Walking velocity > 0.83 m/s in 5 meter walk test |
| Tran 2018 [24]  Canada  (2008-2015) | 19 | N= 40083; 8803 Frail vs 31280 non-frail | 4 +/- 2 years [mean ± sd] | Retrospective (age > 40, primary isolated CABG cases only) | Frail: 1 or more of 10 syndromes in the Hopkins ACG  Non-frail: No syndromes in the Hopkins ACG |

IQR – interquartile range, SD – standard deviation, ASA – American Society of Anesthesiologists, Hopkins ACG – Hopkins Adjusted Clinical Groups, ADL – activities of daily living

Supplementary Table 2: Comparisons of Pooled Outcomes Using All Studies vs Only Studies Using the Same 5 Metre/6 Second Walk Test Frailty Measure

| Outcome | All Studies | Only Studies Using 5 Metre/6 Second Walk Test Frailty Measure with 0.83 m/s Threshold  (Up to 4 studies, 15,468 patients) |
| --- | --- | --- |
| Unadjusted Perioperative Mortality | RR 2.35 (95% CI 1.57-3.51; 14 studies) | RR 2.44 (95% CI 1.82-3.26; 4 studies) |
| Adjusted Perioperative Mortality | Adj OR 1.97 (1.51-2.57; 4 studies) | Adj OR 1.79 (95%CI 1.33-2.42; 1 study) |
|  |  |  |
| Reoperation | RR 1.36 (95% CI 1.15-2.62; 8 studies) | RR 1.27 (95%CI 0.98-1.65; 4 studies) |
| Prolonged (>24-48h) Mechanical Ventilation | RR 2.07 (95% CI 1.86-2.32; 9 studies) | RR 1.75 (95%CI 1.53-2.01; 4 studies) |
| Acute Kidney Injury | RR 2.24 (95% CI 1.85-2.71; 10 studies) | RR 1.91 (95%CI 1.50-2.43; 4 studies) |
| Deep Sternal Wound Infection | RR 2.08 (95% CI 1.14-3.79; 7 studies) | RR 1.82 (95%CI 0.87-3.82; 4 studies) |
| Delirium | RR 2.31 (95% CI 1.16-4.61; 3 studies) | n/a |
| 30d Re-admission | RR 1.19 (95% CI 0.49-2.91; 4 studies) | n/a |
|  |  |  |
| Unadjusted Prolonged Hospital Stay | RR 1.83 (95% CI 1.61-2.08; 5 studies) | RR 2.20 (95% CI 1.30-3.72; 3 studies) |
| Adjusted Prolonged Hospital Stay | Adj OR 2.32 (95% CI 0.95-5.67; 1 study) | Adj OR 2.32 (95% CI 0.95-5.67; 1 study) |
| Unadjusted Discharge to Intermediate Care | RR 2.71 (95% CI 1.45-5.05; 6 studies) | RR 2.17 (95% CI 1.27-3.70; 2 studies) |
| Adjusted Discharge to Intermediate Care | Adj OR 4.61 (95% CI 2.78-7.66; 3 studies) | Adj OR 3.19 (95% CI 1.30-7.82; 1 study) |
|  |  |  |
| Unadjusted Medium-Term Mortality | HR 3.27 (95% CI 1.93-5.55; 10 studies) | n/a |
| Adjusted Medium-Term Mortality | Adj HR 1.37 (95% CI 1.03-1.83; 5 studies) | n/a |
